# Bis(cyclo­hexyl­ammonium) tetra­chlorido­(oxalato)stannate(IV)

**DOI:** 10.1107/S1600536813019284

**Published:** 2013-07-27

**Authors:** Modou Sarr, Aminata Diasse-Sarr, Waly Diallo, Laurent Plasseraud, Hélène Cattey

**Affiliations:** a Laboratoire de Chimie Minérale et Analytique (LACHIMIA), Département de Chimie, Faculté des Sciences et Techniques, Université Cheikh Anta Diop, Dakar, Senegal; b ICMUB UMR 6302, Université de Bourgogne, Faculté des Sciences, 9 avenue Alain Savary, 21000 Dijon, France

## Abstract

The title salt, (C_6_H_14_N)_2_[Sn(C_2_O_4_)Cl_4_], was obtained as a by-product from the reaction between 2C_6_H_14_N^+^·C_2_O_4_^2−^·1.5H_2_O and SnCl_2_·2H_2_O. The cyclo­hexyl­ammonium cation has a chair conformation. The complex anion consists of an oxalate anion chelating the SnCl_4_ moiety, resulting in a distorted octa­hedral coordination sphere of the Sn^IV^ atom with the O atoms in equatorial *cis* positions. In the crystal, cations and anions are linked through N—H⋯O and N—H⋯Cl inter­actions into a layered arrangement parallel to (100).

## Related literature

For applications of organotin(IV) compounds, see: Evans & Karpel (1985 ▶). For background to organotin(IV) chemistry, see: Ballmann *et al.* (2009 ▶); Meriem *et al.* (1989 ▶); Ng & Kumar Das (1997 ▶); Yin & Wang (2004 ▶); Zhang *et al.* (2006 ▶). For background to halogenidotin(IV) chemistry, see: Sarr & Diop (1990 ▶); Qamar-Kane & Diop (2010 ▶); Willey *et al.* (1998 ▶); Diallo *et al.* (2009 ▶). For related crystal structures with an oxalatotin(IV) moiety, see: Skapski *et al.* (1974 ▶); Gueye *et al.* (2012 ▶); Sow *et al.* (2010 ▶, 2013 ▶).
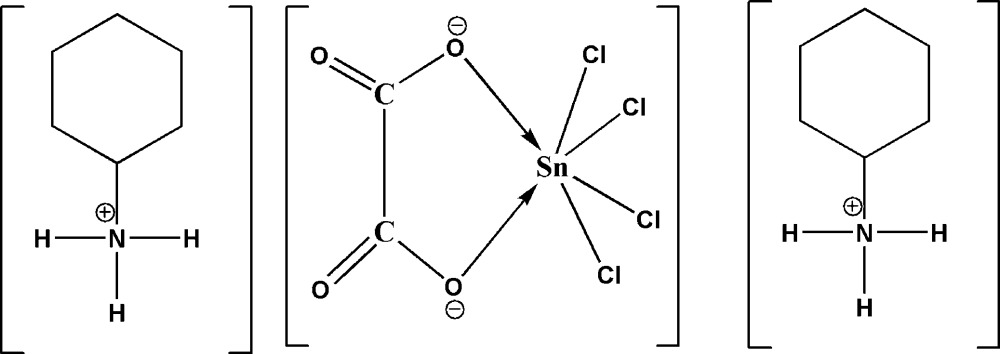


## Experimental

### 

#### Crystal data

(C_6_H_14_N)_2_[Sn(C_2_O_4_)Cl_4_]
*M* *_r_* = 548.87
Monoclinic, 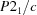

*a* = 11.2293 (9) Å
*b* = 15.715 (1) Å
*c* = 12.8464 (10) Å
β = 93.238 (2)°
*V* = 2263.4 (3) Å^3^
*Z* = 4
Mo *K*α radiation
μ = 1.62 mm^−1^
*T* = 115 K
0.17 × 0.08 × 0.03 mm

#### Data collection

Nonius KappaCCD diffractometer
Absorption correction: multi-scan (Blessing, 1995 ▶) *T*_min_ = 0.770, *T*_max_ = 0.953
9402 measured reflections
4979 independent reflections
4503 reflections with *I* > 2σ(*I*)
*R*_int_ = 0.025

#### Refinement

*R*[*F* ^2^ > 2σ(*F* ^2^)] = 0.035
*wR*(*F* ^2^) = 0.083
*S* = 1.12
4979 reflections
228 parameters
H-atom parameters constrained
Δρ_max_ = 1.03 e Å^−3^
Δρ_min_ = −0.99 e Å^−3^

### 

Data collection: *COLLECT* (Nonius, 1998 ▶); cell refinement: *DENZO-SMN* (Otwinowski & Minor, 1997 ▶); data reduction: *DENZO-SMN*; program(s) used to solve structure: *SIR92* (Altomare *et al.*, 1993 ▶); program(s) used to refine structure: *SHELXL97* (Sheldrick, 2008 ▶); molecular graphics: *ORTEP-3 for Windows* (Farrugia, 2012 ▶) and *Mercury* (Macrae *et al.*, 2006 ▶); software used to prepare material for publication: *WinGX* (Farrugia, 2012 ▶).

## Supplementary Material

Crystal structure: contains datablock(s) global, I. DOI: 10.1107/S1600536813019284/wm2756sup1.cif

Structure factors: contains datablock(s) I. DOI: 10.1107/S1600536813019284/wm2756Isup2.hkl

Additional supplementary materials:  crystallographic information; 3D view; checkCIF report

## Figures and Tables

**Table 1:** Selected bond lengths (Å)

Sn1—O2	2.121 (2)
Sn1—O1	2.155 (2)
Sn1—Cl3	2.3547 (9)
Sn1—Cl2	2.3667 (9)
Sn1—Cl1	2.3794 (9)
Sn1—Cl4	2.4407 (8)

**Table 2:** Hydrogen-bond geometry (Å, °)

*D*—H⋯*A*	*D*—H	H⋯*A*	*D*⋯*A*	*D*—H⋯*A*
N1—H1*A*⋯O4	0.89	1.97	2.853 (3)	169
N1—H1*B*⋯O1^i^	0.89	2.18	3.038 (4)	163
N1—H1*C*⋯O3^ii^	0.89	2.01	2.875 (4)	162
N2—H2*A*⋯O4	0.89	2.25	2.887 (4)	129
N2—H2*A*⋯Cl4^i^	0.89	2.78	3.315 (3)	120
N2—H2*B*⋯Cl4^iii^	0.89	2.38	3.262 (3)	169
N2—H2*C*⋯O3	0.89	2.02	2.869 (4)	158

Symmetry codes: (i) 
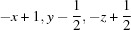
; (ii) 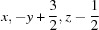; (iii) 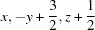.

**Fig. 1.**
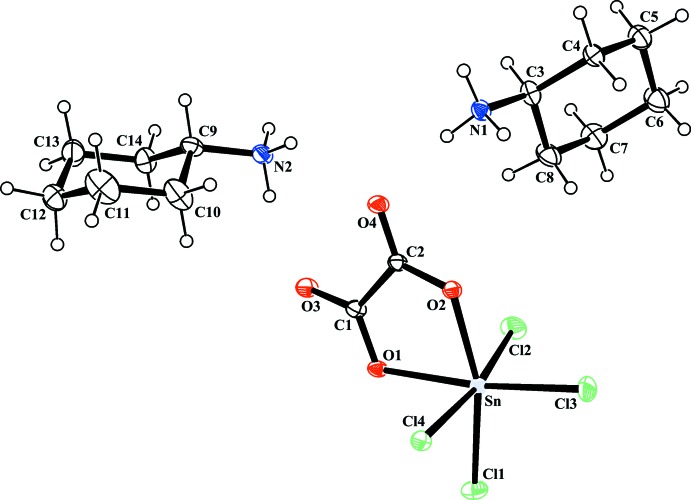
A view of the molecular entities of compound (I) with atom labelling. Displacement ellipsoids are draw at the 30% probability level.

**Fig. 2.**
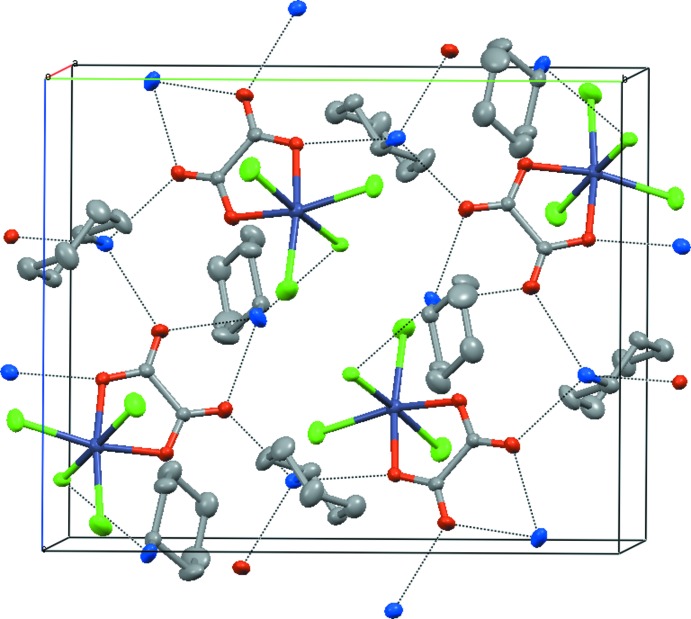
The crystal packing of compound (I) viewed approximately along [100]. Hydrogen atoms are omitted for clarity. Intermolecular hydrogen-bonding interactions of the types N—H···O and O—H···O are shown by dotted lines. Displacement ellipsoids are draw at the 30% probability level. Colour code: Sn dark grey, O red, N blue, Cl green, C grey.

## supplementary crystallographic information

### Comment

Various applications of organotin(IV) compounds exist in many fields, e.g. related to agriculture, medicine, anti-fouling paints and wood preservatives (Evans & Karpel, 1985). Therefore new organotin compounds have been the research subject of many groups (Ballmann *et al.*, 2009; Meriem *et al.*, 1989; Ng & Kumar Das, 1997; Zhang *et al.*, 2006). Our group has previously reported several halogenidotin(IV) derivatives (Diallo *et al.*, 2009; Qamar-Kane & Diop, 2010). In this work, we report the results of the reaction between (C_6_H_14_N)_2_(C_2_O_4_).1.5H_2_O with SnCl_2_.2H_2_O leading to the formation of the title compound, (C_6_H_14_N)_2_[Sn(C_2_O_4_)Cl_4_], (I).

The asymmetric unit of (I) consists of an oxalate anion chelating a SnCl_4_ moiety (Fig. 1). The C—O distances in the oxalate anion [C1—O3 = 1.227 (4) Å; C2—O4 = 1.217 (4) Å; C1—O1 = 1.286 (4) Å; C2—O2 = 1.290 (4) Å] indicate double and single bonds, respectively. The Sn—O distances [Sn1—O1 = 2.155 (2) Å; Sn1—O2 = 2.121 (2) Å] and the Sn—Cl distances [Sn1—Cl1 = 2.3794 (9) Å; Sn1—Cl2 = 2.3667 (9) Å, Sn1—Cl3 = 2.3547 (9) Å and Sn1—Cl4 = 2.4407 (8) Å] (Table 1) are in good agreement with those of previously reported Sn—O and Sn—Cl bonds of related compounds (Willey *et al.*,1998; Skapski *et al.*, 1974; Sow *et al.*, 2010, 2013). The coordination sphere around the Sn^IV^ atom can be described as a slightly distorted octahedron with the O atoms of the oxalate ligand occupying equatorial *cis*-positions. The greatest deviation from the ideal octahedral geometry is manifested in the contraction of the Cl2—Sn1—Cl4 angle to 171.25 (3) °.

The stannate(IV) anion interacts with the two distinct cyclohexylammonium cations (both with chair conformation) through N—H···O and N—H···Cl hydrogen bonds (Table 2), leading to a two-dimensional network extending parallel to (100) (Fig. 2).

### Experimental

Chemicals were purchased from Sigma-Aldrich, and used without further purification. The title compound was obtained by reacting (C_6_H_14_N)_2_(C_2_O_4_).1.5H_2_O (0.09 g, 0.286 mmol) with SnCl_2_.2H_2_O (0,20 g, 0.890 mmol) in 25 ml of ethanol (96%). After slow solvent evaporation of the solvent at room temperature, colourless crystals suitable for X-ray diffraction analysis were obtained.

### Refinement

All H atoms, on carbon and nitrogen atoms, were placed at calculated positions using a riding model with C—H = 0.97 Å (methylene) or 0.98 Å (methine) and N—H = 0.89 Å with *U*_iso_(H) = 1.2*U*_eq_(C) and *U*_iso_(H) = 1.5*U*_eq_(N).

### Crystal data

**Table:**

(C_6_H_14_N)_2_[Sn(C_2_O_4_)Cl_4_]	*F*(000) = 1104
*M**_r_* = 548.87	*D*_x_ = 1.611 Mg m^−^^3^
Monoclinic, *P*2_1_/*c*	Mo *K*α radiation, λ = 0.71073 Å
Hall symbol: -P 2ybc	Cell parameters from 41791 reflections
*a* = 11.2293 (9) Å	θ = 1.0–27.5°
*b* = 15.715 (1) Å	µ = 1.62 mm^−^^1^
*c* = 12.8464 (10) Å	*T* = 115 K
β = 93.238 (2)°	Prism, colourless
*V* = 2263.4 (3) Å^3^	0.17 × 0.08 × 0.03 mm
*Z* = 4	

### Data collection

**Table:**

Nonius KappaCCD diffractometer	4979 independent reflections
Radiation source: fine-focus sealed tube	4503 reflections with *I* > 2σ(*I*)
Graphite monochromator	*R*_int_ = 0.025
φ scans (κ = 0) + additional ω scans	θ_max_ = 27.5°, θ_min_ = 2.7°
Absorption correction: multi-scan (Blessing, 1995)	*h* = −14→10
*T*_min_ = 0.770, *T*_max_ = 0.953	*k* = −20→12
9402 measured reflections	*l* = −16→16

### Refinement

**Table:**

Refinement on *F*^2^	Primary atom site location: structure-invariant direct methods
Least-squares matrix: full	Secondary atom site location: difference Fourier map
*R*[*F*^2^ > 2σ(*F*^2^)] = 0.035	Hydrogen site location: inferred from neighbouring sites
*wR*(*F*^2^) = 0.083	H-atom parameters constrained
*S* = 1.12	*w* = 1/[σ^2^(*F*_o_^2^) + (0.0196*P*)^2^ + 5.6981*P*] where *P* = (*F*_o_^2^ + 2*F*_c_^2^)/3
4979 reflections	(Δ/σ)_max_ = 0.001
228 parameters	Δρ_max_ = 1.03 e Å^−^^3^
0 restraints	Δρ_min_ = −0.99 e Å^−^^3^

### Special details

**Table:**

Geometry. All e.s.d.'s (except the e.s.d. in the dihedral angle between two l.s. planes) are estimated using the full covariance matrix. The cell e.s.d.'s are taken into account individually in the estimation of e.s.d.'s in distances, angles and torsion angles; correlations between e.s.d.'s in cell parameters are only used when they are defined by crystal symmetry. An approximate (isotropic) treatment of cell e.s.d.'s is used for estimating e.s.d.'s involving l.s. planes.
Refinement. Refinement of *F*^2^ against ALL reflections. The weighted *R*-factor *wR* and goodness of fit *S* are based on *F*^2^, conventional *R*-factors *R* are based on *F*, with *F* set to zero for negative *F*^2^. The threshold expression of *F*^2^ > σ(*F*^2^) is used only for calculating *R*-factors(gt) *etc*. and is not relevant to the choice of reflections for refinement. *R*-factors based on *F*^2^ are statistically about twice as large as those based on *F*, and *R*- factors based on ALL data will be even larger.

### Fractional atomic coordinates and isotropic or equivalent isotropic displacement parameters (Å^2^)

**Table:**

	*x*	*y*	*z*	*U*_iso_*/*U*_eq_	
Sn1	0.67656 (2)	0.922515 (13)	0.212827 (17)	0.02314 (7)	
C1	0.5332 (3)	0.8427 (2)	0.3693 (2)	0.0240 (6)	
C2	0.5332 (3)	0.77851 (19)	0.2768 (2)	0.0222 (6)	
O1	0.5778 (2)	0.91628 (13)	0.35167 (16)	0.0231 (5)	
O2	0.5826 (2)	0.80588 (14)	0.19492 (17)	0.0248 (5)	
O3	0.4905 (2)	0.82201 (15)	0.45140 (17)	0.0326 (6)	
O4	0.4875 (2)	0.70888 (14)	0.28564 (17)	0.0282 (5)	
Cl1	0.74421 (8)	1.05949 (5)	0.26855 (7)	0.0349 (2)	
Cl2	0.83254 (8)	0.84828 (6)	0.30516 (8)	0.0403 (2)	
Cl3	0.75536 (10)	0.91018 (6)	0.04748 (8)	0.0428 (2)	
Cl4	0.49591 (7)	0.98965 (5)	0.13685 (6)	0.02334 (16)	
N1	0.5547 (2)	0.58436 (17)	0.1384 (2)	0.0254 (6)	
H1A	0.5344	0.6179	0.1904	0.038*	
H1B	0.5045	0.5406	0.1325	0.038*	
H1C	0.5515	0.6138	0.0792	0.038*	
C3	0.6796 (3)	0.5518 (2)	0.1610 (3)	0.0317 (7)	
H3	0.6778	0.5129	0.2204	0.038*	
C4	0.7205 (3)	0.5014 (2)	0.0690 (3)	0.0305 (7)	
H4A	0.7208	0.5379	0.0081	0.037*	
H4B	0.6654	0.4550	0.0536	0.037*	
C5	0.8453 (4)	0.4659 (3)	0.0929 (3)	0.0397 (9)	
H5A	0.8435	0.4252	0.1496	0.048*	
H5B	0.8720	0.4365	0.0321	0.048*	
C6	0.9310 (4)	0.5365 (3)	0.1229 (4)	0.0478 (11)	
H6A	0.9388	0.5738	0.0636	0.057*	
H6B	1.0088	0.5124	0.1415	0.057*	
C7	0.8886 (4)	0.5888 (3)	0.2157 (3)	0.0439 (10)	
H7A	0.8893	0.5532	0.2774	0.053*	
H7B	0.9428	0.6360	0.2299	0.053*	
C8	0.7630 (3)	0.6227 (3)	0.1914 (3)	0.0418 (9)	
H8A	0.7352	0.6516	0.2522	0.050*	
H8B	0.7641	0.6637	0.1349	0.050*	
N2	0.3881 (3)	0.65674 (19)	0.4780 (2)	0.0357 (7)	
H2A	0.3940	0.6415	0.4118	0.054*	
H2B	0.4276	0.6197	0.5194	0.054*	
H2C	0.4191	0.7084	0.4880	0.054*	
C9	0.2600 (3)	0.6577 (2)	0.5030 (3)	0.0305 (7)	
H9	0.2261	0.6012	0.4884	0.037*	
C10	0.1935 (4)	0.7220 (3)	0.4339 (3)	0.0507 (12)	
H10A	0.2013	0.7073	0.3613	0.061*	
H10B	0.2274	0.7781	0.4459	0.061*	
C11	0.0621 (5)	0.7226 (4)	0.4580 (4)	0.0613 (14)	
H11A	0.0208	0.7657	0.4157	0.074*	
H11B	0.0271	0.6679	0.4395	0.074*	
C12	0.0451 (4)	0.7404 (3)	0.5710 (4)	0.0463 (10)	
H12A	−0.0388	0.7354	0.5841	0.056*	
H12B	0.0699	0.7983	0.5871	0.056*	
C13	0.1164 (4)	0.6793 (3)	0.6413 (4)	0.0460 (10)	
H13A	0.0836	0.6225	0.6326	0.055*	
H13B	0.1095	0.6960	0.7134	0.055*	
C14	0.2475 (3)	0.6780 (3)	0.6171 (3)	0.0357 (8)	
H14A	0.2831	0.7330	0.6333	0.043*	
H14B	0.2892	0.6355	0.6599	0.043*	

### Atomic displacement parameters (Å^2^)

**Table:**

	*U*^11^	*U*^22^	*U*^33^	*U*^12^	*U*^13^	*U*^23^
Sn1	0.02712 (12)	0.02014 (12)	0.02230 (12)	0.00106 (8)	0.00262 (8)	0.00254 (8)
C1	0.0337 (17)	0.0208 (15)	0.0172 (14)	0.0033 (13)	0.0001 (12)	0.0019 (11)
C2	0.0309 (17)	0.0183 (14)	0.0176 (14)	0.0010 (12)	0.0023 (12)	−0.0001 (11)
O1	0.0344 (12)	0.0175 (10)	0.0173 (10)	0.0006 (9)	0.0015 (9)	−0.0013 (8)
O2	0.0372 (13)	0.0199 (11)	0.0180 (10)	−0.0011 (9)	0.0073 (9)	−0.0024 (8)
O3	0.0566 (17)	0.0251 (12)	0.0170 (11)	−0.0004 (11)	0.0104 (11)	−0.0013 (9)
O4	0.0425 (14)	0.0202 (11)	0.0227 (11)	−0.0028 (10)	0.0078 (10)	−0.0031 (9)
Cl1	0.0358 (5)	0.0249 (4)	0.0430 (5)	−0.0060 (3)	−0.0077 (4)	0.0024 (3)
Cl2	0.0355 (5)	0.0359 (5)	0.0487 (5)	0.0104 (4)	−0.0055 (4)	0.0070 (4)
Cl3	0.0523 (6)	0.0416 (5)	0.0369 (5)	0.0105 (4)	0.0239 (4)	0.0083 (4)
Cl4	0.0294 (4)	0.0237 (4)	0.0168 (3)	0.0014 (3)	0.0000 (3)	0.0001 (3)
N1	0.0298 (15)	0.0261 (14)	0.0206 (13)	0.0021 (11)	0.0031 (11)	−0.0023 (10)
C3	0.0303 (18)	0.0380 (19)	0.0271 (17)	0.0054 (15)	0.0022 (14)	−0.0047 (14)
C4	0.0336 (18)	0.0315 (18)	0.0265 (17)	0.0019 (14)	0.0022 (14)	−0.0053 (14)
C5	0.037 (2)	0.039 (2)	0.043 (2)	0.0051 (17)	0.0044 (17)	−0.0049 (17)
C6	0.034 (2)	0.046 (2)	0.063 (3)	0.0009 (18)	0.004 (2)	−0.015 (2)
C7	0.034 (2)	0.049 (2)	0.047 (2)	−0.0044 (18)	−0.0078 (18)	−0.0111 (19)
C8	0.038 (2)	0.038 (2)	0.049 (2)	−0.0018 (17)	0.0006 (18)	−0.0200 (18)
N2	0.054 (2)	0.0232 (14)	0.0315 (15)	0.0092 (13)	0.0196 (14)	0.0088 (12)
C9	0.042 (2)	0.0235 (16)	0.0261 (16)	0.0048 (14)	0.0021 (15)	0.0010 (13)
C10	0.071 (3)	0.057 (3)	0.0249 (18)	0.030 (2)	0.0033 (19)	0.0093 (18)
C11	0.059 (3)	0.072 (3)	0.051 (3)	0.024 (3)	−0.022 (2)	0.000 (2)
C12	0.038 (2)	0.044 (2)	0.058 (3)	0.0096 (18)	0.0054 (19)	0.005 (2)
C13	0.042 (2)	0.049 (2)	0.048 (2)	0.0089 (19)	0.0129 (19)	0.012 (2)
C14	0.039 (2)	0.045 (2)	0.0228 (17)	0.0058 (17)	0.0025 (15)	0.0030 (15)

### Geometric parameters (Å, º)

**Table:**

Sn1—O2	2.121 (2)	C7—C8	1.524 (6)
Sn1—O1	2.155 (2)	C7—H7A	0.9700
Sn1—Cl3	2.3547 (9)	C7—H7B	0.9700
Sn1—Cl2	2.3667 (9)	C8—H8A	0.9700
Sn1—Cl1	2.3794 (9)	C8—H8B	0.9700
Sn1—Cl4	2.4407 (8)	N2—C9	1.491 (5)
C1—O3	1.227 (4)	N2—H2A	0.8900
C1—O1	1.286 (4)	N2—H2B	0.8900
C1—C2	1.559 (4)	N2—H2C	0.8900
C2—O4	1.217 (4)	C9—C10	1.514 (5)
C2—O2	1.290 (4)	C9—C14	1.514 (5)
N1—C3	1.505 (4)	C9—H9	0.9800
N1—H1A	0.8900	C10—C11	1.524 (7)
N1—H1B	0.8900	C10—H10A	0.9700
N1—H1C	0.8900	C10—H10B	0.9700
C3—C8	1.493 (5)	C11—C12	1.501 (7)
C3—C4	1.515 (5)	C11—H11A	0.9700
C3—H3	0.9800	C11—H11B	0.9700
C4—C5	1.524 (5)	C12—C13	1.515 (6)
C4—H4A	0.9700	C12—H12A	0.9700
C4—H4B	0.9700	C12—H12B	0.9700
C5—C6	1.504 (6)	C13—C14	1.521 (5)
C5—H5A	0.9700	C13—H13A	0.9700
C5—H5B	0.9700	C13—H13B	0.9700
C6—C7	1.544 (6)	C14—H14A	0.9700
C6—H6A	0.9700	C14—H14B	0.9700
C6—H6B	0.9700		
			
O2—Sn1—O1	76.97 (8)	C8—C7—H7A	109.6
O2—Sn1—Cl3	92.35 (6)	C6—C7—H7A	109.6
O1—Sn1—Cl3	168.53 (7)	C8—C7—H7B	109.6
O2—Sn1—Cl2	88.73 (7)	C6—C7—H7B	109.6
O1—Sn1—Cl2	87.92 (6)	H7A—C7—H7B	108.1
Cl3—Sn1—Cl2	96.10 (4)	C3—C8—C7	110.6 (3)
O2—Sn1—Cl1	164.34 (6)	C3—C8—H8A	109.5
O1—Sn1—Cl1	87.86 (6)	C7—C8—H8A	109.5
Cl3—Sn1—Cl1	102.47 (4)	C3—C8—H8B	109.5
Cl2—Sn1—Cl1	94.62 (3)	C7—C8—H8B	109.5
O2—Sn1—Cl4	86.17 (7)	H8A—C8—H8B	108.1
O1—Sn1—Cl4	84.01 (6)	C9—N2—H2A	109.5
Cl3—Sn1—Cl4	91.21 (3)	C9—N2—H2B	109.5
Cl2—Sn1—Cl4	171.25 (3)	H2A—N2—H2B	109.5
Cl1—Sn1—Cl4	88.47 (3)	C9—N2—H2C	109.5
O3—C1—O1	124.4 (3)	H2A—N2—H2C	109.5
O3—C1—C2	120.1 (3)	H2B—N2—H2C	109.5
O1—C1—C2	115.5 (3)	N2—C9—C10	109.3 (3)
O4—C2—O2	125.4 (3)	N2—C9—C14	110.7 (3)
O4—C2—C1	119.5 (3)	C10—C9—C14	110.9 (3)
O2—C2—C1	115.1 (3)	N2—C9—H9	108.6
C1—O1—Sn1	114.25 (19)	C10—C9—H9	108.6
C2—O2—Sn1	115.71 (19)	C14—C9—H9	108.6
C3—N1—H1A	109.5	C9—C10—C11	109.7 (4)
C3—N1—H1B	109.5	C9—C10—H10A	109.7
H1A—N1—H1B	109.5	C11—C10—H10A	109.7
C3—N1—H1C	109.5	C9—C10—H10B	109.7
H1A—N1—H1C	109.5	C11—C10—H10B	109.7
H1B—N1—H1C	109.5	H10A—C10—H10B	108.2
C8—C3—N1	111.1 (3)	C12—C11—C10	112.0 (4)
C8—C3—C4	112.4 (3)	C12—C11—H11A	109.2
N1—C3—C4	110.4 (3)	C10—C11—H11A	109.2
C8—C3—H3	107.6	C12—C11—H11B	109.2
N1—C3—H3	107.6	C10—C11—H11B	109.2
C4—C3—H3	107.6	H11A—C11—H11B	107.9
C3—C4—C5	110.5 (3)	C11—C12—C13	111.5 (4)
C3—C4—H4A	109.6	C11—C12—H12A	109.3
C5—C4—H4A	109.6	C13—C12—H12A	109.3
C3—C4—H4B	109.6	C11—C12—H12B	109.3
C5—C4—H4B	109.6	C13—C12—H12B	109.3
H4A—C4—H4B	108.1	H12A—C12—H12B	108.0
C6—C5—C4	110.4 (3)	C12—C13—C14	111.8 (3)
C6—C5—H5A	109.6	C12—C13—H13A	109.3
C4—C5—H5A	109.6	C14—C13—H13A	109.3
C6—C5—H5B	109.6	C12—C13—H13B	109.3
C4—C5—H5B	109.6	C14—C13—H13B	109.3
H5A—C5—H5B	108.1	H13A—C13—H13B	107.9
C5—C6—C7	111.7 (4)	C9—C14—C13	110.2 (3)
C5—C6—H6A	109.3	C9—C14—H14A	109.6
C7—C6—H6A	109.3	C13—C14—H14A	109.6
C5—C6—H6B	109.3	C9—C14—H14B	109.6
C7—C6—H6B	109.3	C13—C14—H14B	109.6
H6A—C6—H6B	107.9	H14A—C14—H14B	108.1
C8—C7—C6	110.5 (3)		

### Hydrogen-bond geometry (Å, º)

**Table:**

*D*—H···*A*	*D*—H	H···*A*	*D*···*A*	*D*—H···*A*
N1—H1*A*···O4	0.89	1.97	2.853 (3)	169
N1—H1*B*···O1^i^	0.89	2.18	3.038 (4)	163
N1—H1*C*···O3^ii^	0.89	2.01	2.875 (4)	162
N2—H2*A*···O4	0.89	2.25	2.887 (4)	129
N2—H2*A*···Cl4^i^	0.89	2.78	3.315 (3)	120
N2—H2*B*···Cl4^iii^	0.89	2.38	3.262 (3)	169
N2—H2*C*···O3	0.89	2.02	2.869 (4)	158

Symmetry codes: (i) −*x*+1, *y*−1/2, −*z*+1/2; (ii) *x*, −*y*+3/2, *z*−1/2; (iii) *x*, −*y*+3/2, *z*+1/2.
